# LIV selection in ‘tweener’ patients treated with magnetically controlled growing rods vs. posterior spinal fusion

**DOI:** 10.1007/s43390-024-01019-2

**Published:** 2024-12-15

**Authors:** Brandon Yoshida, Claudia Leonardi, Jacquelyn Valenzuela-Moss, Lindsay M. Andras, Tyler A. Tetreault, John B. Emans, John T. Smith, Joshua M. Pahys, G. Ying Li, Pediatric Spine Study Group, Michael J. Heffernan

**Affiliations:** 1 Keck School of Medicine, University of Southern California, Los Angeles, CA USA; 2 School of Public Health, LSU Health Sciences Center, New Orleans, LA USA; 3 Children’s Hospital Los Angeles, Jackie and Gene Autry Orthopedic Center, Los Angeles, CA USA; 4 Orthopedics and Sports Medicine Department, Boston Children’s Hospital, Boston, MA USA; 5 Department of Orthopaedics, The Orthopaedic Partners, Park City, UT USA; 6 Pediatric Orthopedics Department, Shriners Hospital for Children, Philadelphia, PA USA; 7 Department of Orthopaedic Surgery, C.S. Mott Children’s Hospital, Ann Arbor, MI USA

**Keywords:** Lowest instrumented vertebrae, Growth friendly instrumentation, LIV selection, ‘Tweener’ patients, Posterior spinal fusion

## Abstract

**Purpose:**

The purpose of this study was to compare the LIV selection in ‘tweener’ patients treated with MCGR or PSF.

**Methods:**

A multicenter pediatric spine database was queried for ambulatory patients ages 8–11 years treated by MCGR or PSF with at least 2-year follow-up. The relationship between the LIV and preoperative spinal height, curve magnitude, and implant type were assessed. The relationship between the touched vertebrae (TV), the last substantially touched vertebrae (LSTV), the stable vertebrae (SV), and the LIV were evaluated.

**Results:**

One hundred and fifty-nine patients met inclusion criteria. Preoperative curve magnitude was similar between groups (MCGR 68 ± 19.0° vs. PSF 66 ± 17.2°, *p* = 0.6). Preoperative curve magnitude was associated with LIV, as larger curves were associated with a more caudal LIV (*p* = 0.0004). Distribution of the LIV was more varied in PSF compared to MCGR. L3 was the LIV in 43% of MCGR patients compared to 27% of PSF patients. A thoracic LIV was more common in the PSF group (PSF 13% vs. MCGR 1.2%, *p* = 0.0038). The LIV was cephalad to the SV in 68% of PSF compared to 48% of MCGR patients (*p* = 0.02).

**Conclusion:**

The majority of LIV selection in ‘tweener’ patients was at L3 or below regardless of surgical strategy, likely driven by curve magnitude. However, ‘tweener’ patients treated with PSF had more cephalad LIV selections compared to patients treated with MCGR. Potential LIV differences should be considered when selecting MCGR vs. PSF in ‘tweener’ patients.

**Level of evidence:**

III.

## Introduction

Early onset scoliosis (EOS) is a complex spinal deformity in children with a curvature > 10° and onset less than 10 years of age [1]. Patient age and remaining growth play a large role in selection of surgical strategy for pediatric scoliosis. Growth friendly instrumentation, such as magnetically controlled growing rods (MCGR), are commonly utilized for the treatment of young children with EOS [2]. In contrast, posterior spinal fusion (PSF) is the mainstay of treatment for skeletally mature patients. There is significant equipoise regarding the ideal treatment of patients with EOS between ages 8 and 11 years, often referred to as ‘tweeners,’ a group where both MCGR and PSF are utilized [3]. For either strategy, one critical consideration is the lowest instrumented vertebra (LIV). The LIV is the last vertebra included in the instrumentation construct and its selection can significantly impact lower back flexibility and risk of disk degeneration in adulthood [4, 5].

Despite the widespread use of MCGR and PSF in the management of ‘tweener’ patients with EOS, to our knowledge, there are no prior studies directly comparing the LIV between the two techniques. The purpose of this study was to compare the LIV selection in ‘tweener’ patients whose index procedure was MCGR vs. PSF. We hypothesized that patients treated with MCGR would have more caudal LIV selection when compared to PSF due to the need to accommodate the rigid portion of the MCGR actuator.

## Methods

This was a retrospective review of an institutional review board-approved, multicenter international database of patients diagnosed with EOS and treated with MCGR or PSF.

### Eligibility and selection criteria

Ambulatory EOS patients with an idiopathic diagnosis between the ages of 8 and 11 years treated with MCGR or PSF between 2010 and 2020 with minimum 2-year follow-up were screened for eligibility. Patients with incomplete data, instrumentation extending to S1 or below, or listed as having had prior surgical instrumentation were excluded from the study.

### Data collection

Baseline patient characteristics collected included age, sex, race, weight, and height. Pre-index data included major curve magnitude, thoracic spine height (TSH) from T1–T12 and lumbar spine height from L1–S1; TSH was defined as the vertical distance between true horizontal (0°) lines from the superior endplate of T1 to the superior endplate of L1 on calibrated images. Lumbar spine height was measured similarly between the L1 and S1 superior endplates. All images were calibrated utilizing the radiographic measurement standard operating procedures of the Pediatric Spine Study Group. Operative details including date of surgery and instrumentation type (MCGR vs. PSF) were recorded. A center sacral vertical line (CSVL) was drawn on upright preoperative anterior–posterior radiographs to assess the last touched vertebrae (TV), the last substantially touched vertebrae (LSTV), and the stable vertebrae (SV). The TV was defined as the most cephalad thoracolumbar or lumbar vertebra that was “touched” by the CSVL on any portion of the involved vertebrae. The LSTV was defined as the most cephalad thoracolumbar or lumbar vertebrae that the CSVL at least touched or was medial to the lateral border of the pedicle. The SV was defined as the most cephalad vertebrae below the curve apex that was most closely bisected by the CSVL. The primary outcome was the LIV, which was defined as the most caudal instrumented vertebrae as determined by postoperative radiographs. Secondary outcomes included factors associated with the LIV as well as the relationship between the TV, LSTV, SV, and the LIV.

### Study design

The LIV was assessed in relation to preoperative spinal height, curve magnitude, and implant type. We then compared the relationship between TV, LSTV, SV, and the LIV in patients treated with MCGR and PSF.

### Data analysis

Data were analyzed using SAS/STAT software version 9.4 (SAS Institute Inc., Cary, NC). The association between LIV and selected patient demographics and clinical characteristics within lumbar vertebrae was compared using ANOVA and Tukey adjustment for multiple comparisons when an overall significant association was observed. The LIV was compared between surgical instrumentation type (MCGR vs. PSF) using the *χ*^2^ test for categorical variables. Where applicable, residuals were independent with an identical and normal distribution and homogenous variances. A two-sided *p* < 0.05 indicated statistical significance**.**

## Results

One hundred fifty-nine patients met the inclusion criteria, including 82 treated with MCGR and 77 treated with PSF. Demographics and preoperative measurements are outlined in Table 1. Mean age for the overall cohort was 9.6 ± 0.8 years. Importantly, preoperative curve magnitude was similar between groups (MCGR: 68.0 ± 19.0°; PSF: 66.4 ± 17.2°). However, several demographic characteristics including age (PSF: 10.2 ± 0.8 years; MCGR: 9 ± 1.0 years, *p* < 0.0001), weight (PSF: 40.9 ± 12.5 kg; MCGR: 30.4 ± 8.5 kg, *p* < 0.0001), height (PSF: 144.4 ± 11.3 cm; MCGR: 133 ± 12.4 cm, *p* < 0.0001), and the proportion of females (PSF: 88.3%; MCGR: 68.3%, *p* value = 0.002) were significantly greater in the PSF group compared to the MCGR group. The pre index thoracic height (*p* = 0.04) and lumbar spine height (*p* = 0.23) were also significantly greater in the PSF group.

**Table 1:** Patient demographics and clinical characteristics

Characteristics	Fusion (*n* = 77)	MCGR (*n* = 82)	*p* value
Age (years), mean (SD)	10.2 (0.8)	9.0 (1.0)	< 0.0001
Weight (kg), mean (SD)	40.9 (12.5)	30.4 (8.5)	< 0.0001
Height (cm), mean (SD)	144.4 (11.3)	133.0 (12.4)	< 0.0001
Pre-surgery major Cobb angle (°), mean (SD)	66.4 (17.2)	68.0 (19.0)	0.601
Pre- index thoracic spine height (T1–T12, mm), mean (SD)	206.4 (31.7)	194.5 (29.8)	0.041
Pre- index lumbar spine height (L1–S1, mm), mean (SD)	125.8 (19.5)	117.4 (19.6)	0.023
Sex, % (*n*)			0.002
Female	88.3 (68)	68.3 (56)	
Male	11.7 (9)	31.7 (26)	
Race, % (*n*)			0.157
Black/African American	9.1 (7)	20.7 (17)	
White/Caucasian	64.9 (50)	61.0 (50)	
Other	14.3 (11)	12.2 (10)	
Unknown	11.7 (9)	6.1 (5)	

*SD* standard deviation

**Fig. 1:**
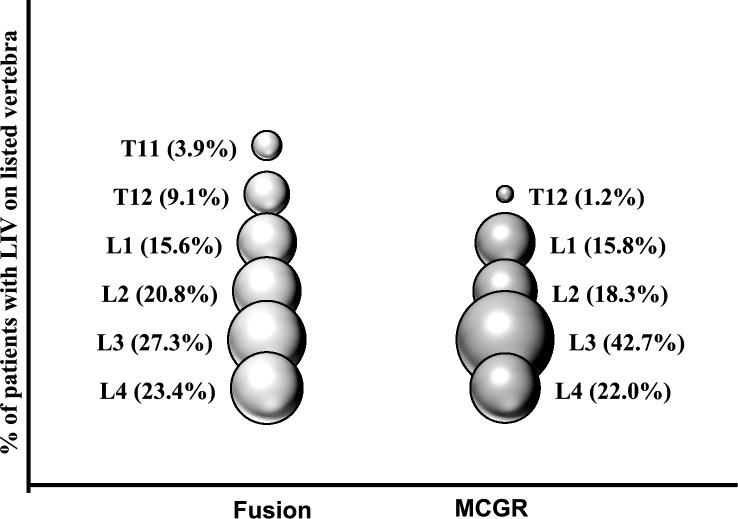
Percentage of patients (bubble width) with lowest instrumented vertebrae (LIV) on listed vertebra by surgery type [fusion vs. magnetically controlled growing rods (MCGR)]

LIV selection was evaluated in relation to patient and curve characteristics (Table 2). Several factors including patient age, height, and radiographically measured preoperative thoracic height were not associated with the LIV (*p* > 0.05). L3 was the most common LIV in both groups. Within the overall cohort, patients with higher curve magnitude were more likely to have a lower LIV (*p* = 0.0004), a finding that was maintained in the MCGR group (*p* = 0.001), but not the PSF group (*p* = 0.123). The distribution of LIV selection (Fig. 1) was different between treatment groups. The PSF group had a wider distribution of LIV selection compared to MCGR. Further, there was a predominance of LIV selection at L3 within the MCGR group (42.7%) that was not seen in the PSF group (27.3%). Sixty five% of patients treated with MCGR had an LIV at L3 or below compared to 50% of the patients treated with PSF. A thoracic LIV was more common in the PSF group (PSF 13% vs. MCGR 1.2%, *p* = 0.0038).

**Table 2:** Patient demographics and clinical characteristics within lumbar vertebrae by LIV

Characteristics	L1 (*n* = 25)	L2 (*n* = 31)	L3 (*n* = 56)	L4 (*n* = 36)	*p* value
All patients					
Age (years), mean (SD)	9.4 (1.0)	9.9 (1.0)	9.4 (1.1)	9.8 (0.2)	0.072
Weight (kg), mean (SD)	32.6 (9.5)	36.0 (10.4)	33.6 (11.4)	37.4 (13.7)	0.307
Height (cm), mean (SD)	135.0 (11.7)	140.1 (11.8)	136.3 (12.2)	140.0 (14.9)	0.270
Pre- surgery major Cobb angle (°), mean (SD)	61 (13.1)	58 (10.0)	73 (20.8)	71 (19.1)	0.0004
Pre- index thoracic spine height (T1–T12, mm), mean (SD)	202.4 (26.2)	205.2 (18.2)	191.1 (29.6)	206.7 (43.4)	0.154
Pre- index thoracic spine height (L1–S1, mm), mean (SD)	124.7 (15.7)	118.7 (12.4)	117.7 (23.2)	122.1 (19.9)	0.556
Fusion	(*n* = 12)	(*n* = 16)	(*n* = 21)	(*n* = 18)	
Age (years), mean (SD)	10.0 (0.7)	10.5 (0.7)	10.0 (0.8)	10.4 (0.8)	0.103
Weight (kg), mean (SD)	37.5 (8.2)	41.9 (10.1)	38.6 (14.4)	43.4 (13.8)	0.504
Height (cm), mean (SD)	142.3 (8.5)	145.7 (10.7)	140.6 (11.5)	147.1 (10.4)	0.235
Pre- surgery major Cobb angle (°), mean (SD)	65.2 (14.4)	59.3 (9.2)	74.2 (21.5)	64.9 (18.8)	0.123
Pre- index thoracic spine height (T1–T12, mm), mean (SD)	201.0 (25.1)	206.2 (17.0)	196.6 (37.9)	222.6 (33.8)	0.214
Pre- index thoracic spine height (L1–S1, mm), mean (SD)	125.3 (14.6)	120.1 (10.4)	126.0 (16.1)	125.3 (16.2)	0.933
MCGR	(*n* = 13)	(*n* = 15)	(*n* = 35)	(*n* = 18)	
Age (years), mean (SD)	8.9 (0.9)	9.3 (1.0)	9.0 (1.0)	9.1 (1.1)	0.691
Weight (kg), mean (SD)	28.2 (8.6)	30.1 (6.7)	30.6 (7.9)	31.5 (10.9)	0.759
Height (cm), mean (SD)	128.2 (10.3)	134.4 (10.5)	133.6 (12.0)	133.3 (15.6)	0.533
Pre- surgery major Cobb angle (°), mean (SD)	58.2^bc^ (11.8)	56.0^c^ (10.7)	72.9^ab^ (20.7)	76.1^a^ (18.4)	0.001
Pre- index thoracic spine height (T1–T12, mm), mean (SD)	203.6 (28.1)	204.5 (19.8)	187.6 (22.6)	192.1 (47.4)	0.287
Pre- index thoracic spine height (L1–S1, mm), mean (SD)	124.2 (17.3)	117.7 (14.1)	112.5 (18.8)	119.3 (23.1)	0.333

*SD* standard deviation, *TSH* thoracic spine height

^a, b, c^Means with different superscripts are significantly different (*p* < 0.05)

**Fig. 2:**
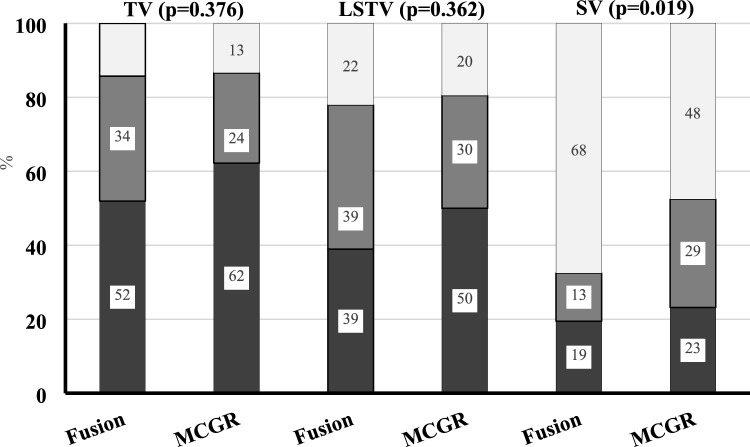
Percentage of patients with Lowest instrumented vertebrae (LIV) proximal (white), at (light gray) or caudal (dark gray) compared to either last touched vertebra (TV), or last substantially touched vertebra (LSTV), or stable vertebra (SV)

The LIV was also evaluated in relation to the TV, LSTV, and the SV (Fig. 2). Although not statistically significant, the LIV trended lower in relation to both the TV and the LSTV in the MCGR group when compared to the PSF group. The LIV was below the TV in 62% of the MCGR group compared to 52% in the PSF group. Similarly, 50% of LIV selections were below the LSTV in the MCGR group compared to 39% of the PSF group. The LIV was cephalad to the SV in 68% of PSF compared to 48% of MCGR patients (*p* = 0.19).

## Discussion

To our knowledge, this study is the first to investigate the LIV in ‘tweener’ EOS patients treated with MCGR and PSF. We found that L3 was the most common LIV regardless of surgical strategy. Curve magnitude seemed to have the strongest influence on LIV selection, particularly in the MCGR group, as larger curves were associated with a more caudal LIV. The LIV selections in the PSF group were both more varied and more cephalad when compared to MCGR which demonstrated a predominance of L3 as the selected LIV. Although selective thoracic surgery was rare in both groups, patients treated with PSF were more likely to have a thoracic LIV when compared to the MCGR group.

The selection of the LIV is a critical aspect of surgical planning in patients with EOS. An appropriately selected LIV helps facilitate optimal spinal balance, maximizes spinal growth potential, and is cost effective, both with respect to implant costs, operating room utilization, and decreased risk of complications and revision surgery [6, 7]. The ‘adding-on’ phenomenon, resulting in increased deviation from the CSVL or increased angulation between the LIV and the uninstrumented vertebra can result from an LIV selection that is too cephalad leading to addition of caudal vertebrae in future revision surgeries [8]. Conversely, an LIV that is too caudal risks decreased lumbar spine flexibility [4, 9, 10], especially if the construct extends below L3 [11]. Furthermore, degenerative disk disease (DDD) is a significant risk factor for patients whose constructs extend into the lumbar spine, with a 27% increased risk of significant DDD with an LIV of L4 compared to more cephalad levels [5]. Therefore, considerate LIV selection is paramount to optimize surgical outcomes and minimize complications.

In our cohort, the most common LIV was L3 for both the MCGR and PSF groups. Although few studies exist comparing PSF to MCGR, some cohort studies investigating MCGR reveal a common LIV of L3 and L4 [12, 13]. On the surface, our results suggest that this is predominantly driven by curve magnitude. However, when we analyzed the LIV within treatment groups, curve magnitude only remained significant in the MCGR group. Further, essentially 50 % of LIV selections were at L3 in the MCGR group compared to only 27% in the PSF group. Similarly, over 50% of LIV selection was at or below the SV in MCGR patients compared to only 30% of the PSF group. Our data indicate a trend toward more caudal LIV selections within the MCGR group when compared to the PSF group. Curve magnitude was similar between groups and therefore is unlikely to explain the differences in LIV selection. While spinal height was different between the MCGR and PSF groups, it is unlikely that the difference in spinal height alone accounts for the LIV selections. Although not explicitly answered by our data, we theorize that this difference could be explained by the need to accommodate the rigid actuator at the thoracolumbar junction in the MCGR group.

In the overall cohort, selective thoracic surgery was uncommon. In fact, 99% of the MCGR group had a lumbar LIV, most commonly at L3. Within the group treated with MCGR, selective thoracic surgery was essentially nonexistent. Although rare, there was a subset of PSF patients who had a selective thoracic fusion. In previous studies, long-term follow-up has demonstrated stable outcomes in appropriately indicated patients [14, 15]. In addition, numerous benefits exist that support the promotion of selective thoracic fusion whenever possible given its decreased pain and increased patient satisfaction, function, and self-image scores compared to fusions to the lumbar spine [16, 17]. Although many patients treated for EOS have a lumbar LIV, the long-term outcomes and patient satisfaction of selective thoracic constructs are worthy of consideration, which may not be possible in patients who undergo an index MCGR based on the results of this study.

In the setting of growth friendly surgeries, MCGRs have been touted as a significant evolution in the operative management of early-onset scoliosis, with numerous studies citing cost neutrality and decreased surgical procedures [18, 19] as significant benefits compared to traditional growing rods. However, there is also literature that questions whether the benefits of MCGR outweigh PSF for EOS which is most relevant for the‘tweener’ group. Initially, metallosis and rod failure were a concern with MCGRs [20, 21] but more recent literature has included inferior outcomes and complications compared to PSF. One retrospective review of 130 EOS patients by Mackey et al. [13] reported on EOS patients treated with MCGR, PSF, or vertebral body tether (VBT) and found that unplanned surgeries were significantly greater in the MCGR group compared to PSF or VBT with a hazard ratio of 5.6. Furthermore, correction of the major curve was significantly decreased compared to PSF, which had a 52.2% curve correction compared to 27.4% in the MCGR group. Increased thoracic height is a potential benefit with MCGRs but diminishing returns in thoracic height has been cited after the 4th distraction secondary to tissue scarring and construct stiffening [12], ultimately resulting in only 1–2 cm of additional thoracic height compared to PSF which has not proven to be clinically beneficial [22]. Therefore, the pros and cons of MCGR compared to PSF must be scrutinized before opting for either treatment strategy in patients with EOS. By comparing LIV section in patients treated with MCGR and PSF, our study highlights another important consideration when choosing a surgical strategy for EOS patients.

Our study is not without limitations. Our study focuses on two posterior-based surgical strategies for the treatment of EOS and does not include other constructs. The retrospective nature of our study could lead to the introduction of selection bias and uncontrolled confounding variables in our analysis. There is some heterogeneity in the age, height, weight, and sex of patients who underwent MCGR and PSF. As this was a descriptive study of LIV selection, patient reported outcomes were not included. We also did not have access to the criteria used by each treating surgeon when selecting an LIV. Finally, this study focused on the LIV at the index procedure. We did not have sufficient data to analyze the LIV of MCGR graduate patients, after conversion to PSF at the end of growth friendly treatment, which could have led to an even greater discrepancy in LIV between groups.

To our knowledge, this study is the first to investigate the LIV in ‘tweener’ EOS patients treated with magnetically controlled growing rods and posterior spinal fusion. Patients treated with PSF more frequently had a LIV that was cephalad to the SV and had a higher rate of selective thoracic surgery, which did not occur in patients treated with MCGR in our study. Given the potential long-term implications for patients with more caudal LIV, surgeons should be aware of the differences in LIV selection between EOS treatment options. Although important, we recognize that LIV selection is a single factor among a multitude of considerations when deciding between MCGR and PSF for patients with EOS. Further investigation must be performed to further clarify the equipoise surrounding treatment of EOS ‘tweener’ patients.

## Data Availability

The data that support the findings of this study are available from the corresponding author and/or the Pediatric Spine Study Group upon reasonable request.
